# Familial Hypercholesterolemia and Acute Coronary Syndromes: The Microbiota–Immunity Axis in the New Diagnostic and Prognostic Frontiers

**DOI:** 10.3390/pathogens12040627

**Published:** 2023-04-21

**Authors:** Andrea Piccioni, Elena Niccolai, Gloria Rozzi, Giacomo Spaziani, Christian Zanza, Marcello Candelli, Marcello Covino, Antonio Gasbarrini, Francesco Franceschi, Amedeo Amedei

**Affiliations:** 1 Emergency Medicine Department, Fondazione Policlinico Universitario Agostino Gemelli-IRCCS, Università Cattolica del Sacro Cuore di Roma, 00168 Roma, Italy; 2 Department of Experimental and Clinical Medicine, University of Florence, 50134 Florence, Italy; 3 Foundation “Ospedale Alba-Bra Onlus”, Department of Emergency Medicine, Anesthesia and Critical Care Medicine, Michele and Pietro Ferrero Hospital, 12060 Verduno, Italy; 4 Medical and Surgical Science Department, Fondazione Policlinico Universitario Agostino Gemelli-IRCCS, Università Cattolica del Sacro Cuore di Roma, 00168 Roma, Italy

**Keywords:** familial hypercholesterolemia, LDL, cardiovascular disease, atherosclerosis, coagulation, gut microbiota, acute coronary syndromes

## Abstract

Familial hypercholesterolemia is a common genetic disorder with a propensity towards early onset of atherosclerotic cardiovascular disease (CVD). The main goal of therapy is to reduce the LDL cholesterol and the current treatment generally consists of statin, ezetimibe and PCSK9 inhibitors. Unfortunately, lowering LDL cholesterol may be difficult for many reasons such as the variation of response to statin therapy among the population or the high cost of some therapies (i.e., PCSK9 inhibitors). In addition to conventional therapy, additional strategies may be used. The gut microbiota has been recently considered to play a part in chronic systemic inflammation and hence in CVD. Several studies, though they are still preliminary, consider dysbiosis a risk factor for various CVDs through several mechanisms. In this review, we provide an update of the current literature about the intricate relation between the gut microbiota and the familial hypercholesterolemia.

## 1. Introduction

Cardiovascular diseases (CVD) are still an open public health care issue of the World Health Organization (WHO) since these are the leading causes of death worldwide [1]. CVD encompasses multiple disorders, including platelet hyperactivity, atherosclerosis, hypertension, stroke, hyperlipidemia, and heart failure [2]. Hypercholesterolemia (especially elevated low-density lipoprotein cholesterol or LDLc) is one of the main modifiable risk factors (hypercholesterolemia, obesity, diabetes, hypertension, and smoking) which are estimated to be the cause for more than a half of CVD [3]. The presence of high levels of LDLc predict a much higher risk of CVD such as in individuals affected by familial hypercholesterolemia (FH).

FH is one of the most common inherited metabolic diseases defined by markedly elevated plasma levels of LDLc while off treatment (≥190 mg/dL) and a history of premature atherosclerosis (if heterozygous FH patients are left untreated, it is estimated that 50% of men at the age of 50 years and 30% of women at the age of 60 years, respectively, develop CVD; if homozygous FH patients are left untreated, they develop CVD in early childhood and generally do not survive beyond 30 years) [4] or CVD [5]. Another high-risk cohort encompasses individuals with polygenic hypercholesterolaemia, involving single nucleotide polymorphisms (SNPs) that individually have a minor impact, but when combined can significantly increase LDLc blood levels [6]. FH has been associated with high rates of CVD, stroke and premature death at a very young age [7].

The susceptibility to CVD differs among patients affected by FH, even when LDLc levels are similar, suggesting that a pathogenic role for CVD may be played by other factors [8].

The vast majority of individuals with familial hypercholesterolemia have autosomal dominant mutations of one of three genes: the LDL receptor (*LDLR*), apolipoprotein B (*APOB*) and proprotein convertase subtilisin/Kexin type 9 (*PCSK*) [9,10]. Each of these mutations leads to LDLc elevation [11], which is the primary contributor to CVD in people with FH [12]. Lipid abnormalities have been shown to play a critical role in the major steps of atherosclerosis as the cholesterol accumulates in the foam cells and in the lipid core of the atherosclerotic plaque [13].

As in the case of FH, all patients with chronically high LDLc levels are thought to be at high risk of CVD and need to receive lipid-lowering drugs [14]. However, recent research has raised doubts about the strict relationship between high levels of cholesterol and atherosclerosis [8].

Elevated LDLc values may not be directly associated with premature CVD events. As accumulating literature suggests, LDL do not independently predict atherosclerotic burden in FH and patients, despite the optimal response to the lipid lowering therapy, still experience CVD [15,16]. The idea that there is no association between the severity of atherosclerosis and serum LDLc levels was already gaining ground in 1937, when Lande, K. E. and Sperry, W. M. failed to find a connection between serum cholesterol and the degree of aortic atherosclerosis of any aged men [17].

Based on available evidence, newly predictive scores for FH patients involve also risk factors other than LDLc blood levels (i.e., coagulation factors, lipoprotein (a) (Lp(a)), lifestyle, hypertension, gender, sex, HDL) [18,19,20]. FH patients who die prematurely have higher Lp(a), and/or higher factor VIII, and/or higher fibrinogen, compared to those with a normal lifespan; conversely, their chronic LDLc blood levels do not differ consistently [21]. Therefore, these variables could be described as surrogate biomarkers of CVD risk in FH population.

The gut microbiota is increasingly recognized for its interactions with CVD development and progression (i.e., atherosclerosis, inflammation, obesity, platelet function and plasma lipid abnormalities). In fact, it can alter blood lipid composition as the cholesterolemia, through the release of microbial products [22,23].

Despite the fact that lipid metabolism, inflammation, vascular stiffness, and blood pressure have all been linked to atherosclerosis, recent literature also reveals new potential players, as the composition and the organization of the intestinal microbiota, in the disease’s evolution, possibly leading to innovative treatments.

Hence, in FH patients’ other newly emerging pathways subtend to CVD, despite LDLc blood levels [16].

In this review, we will provide an update of the literature on the role of gut microbiota and other emerging risk factors as potential therapeutic targets for FH.

## 2. Materials and Methods

A literature research was performed in PubMed and Mendeley electronic databases, using the following key words: “familial hypercholesterolemia”, “premature cardiovascular disease”, “LDL”, “gut microbiota and atherosclerosis”, “Lipoprotein a and familial hypercholesterolemia”, “coagulation factors”, “probiotics and familial hypercholesterolemia”, acute coronary syndromes”. Only English-language articles were included, and preference was given to papers published within the last 15 years. We have searched the bibliographies of the selected articles to identify other relevant articles. We have removed inappropriate or not relevant topics considering the specific focus of this review.

## 3. Results

### 3.1. Coronary Artery Disease and Familial Hypercholesterolemia

The initial clinical manifestation in patients affected by familial hypercholesterolemia may be the occurrence of acute coronary syndrome (ACS) (Table 1). ACS refers to a group of disorders characterized by decreased blood flow, including ST-elevation myocardial infarction (STEMI), non-ST elevation myocardial infarction (NSTEMI), and unstable angina. Current literature suggests that the prevalence of FH among individuals who require in-patient care for ACS was about 10 times higher compared with the general population [24]. In a cohort of 105 individuals with very-early-onset coronary artery diseases (<35 years old), the occurrence of FH pathogenic mutations (including *LDLR*, *APOB*, *PCSK9*, *APOE*, *STAP1*, *LIPA*, *LDLRAP1*, *ABCG5*/*8*) was 38.1% [25].

In a clinical trial that was conducted in the United States and involved 1,697,513 ACS hospital admissions, the authors of the study found that participants with FH were younger, had less concomitant conditions and were more likely to experience in-hospital cardiac problems than those without FH. Moreover, they frequently presented with STEMI and have a higher incidence of recurrent ACS [26].

In the YOUNG-MI registry, nearly 1 out of 10 patients with myocardial infarction at young age were affected by FH. Many of them had elevated LDLc at one year despite high-intensity statin therapy after discharge [27]. 

A cohort of 320 individuals who had survived their first STEMI ≤ 35 years of age revealed that one out of five patients had clinical heterozygous FH, who, despite their use of statins, showed a high recurrence of cardiac events [28].

Moreover, even among individuals who are taking high-intensity lipid-lowering medication, the risk of long-term death is twice as high in FH patients as it is in controls [29].

The Dutch Lipid Clinic Network method is advised by the International Familial Hypercholesterolemia Foundation and the National Lipid Association in the USA to detect FH patients early in order to prevent premature ACS [30,31].

Hence, it becomes clear that an early diagnosis is necessary and new therapeutic targets need to be found.

### 3.2. Coagulation Factors, Lipoprotein (a) and Familial Hypercholesterolemia

In addition to blood levels of LDL cholesterol, other inherited variables may contribute to cardiovascular risk in people with FH, such as coagulation factors and Lp(a) (Table 2). It was found that maternal FH may influence coagulation and fibrinolytic factors in offspring independently of the children’s FH status [32]. Increased coagulation factors are associated with premature cardiovascular diseases in patients affected by FH [21]. The platelets aggregation ability of these people seems to be enhanced compared to those without FH [33]. In research that included 164 patients with FH and 160 control patients, mean platelet volume (MPV) was higher in individuals with FH and was independently correlated with total cholesterol level [34]. It has been shown that larger platelets are more reactive and more prone to adhesion and that MPV may predict CVD and outcomes in patients with coronary artery disease (CAD) [35]. Another important parameter is factor VII (FVII), that has a key role in coagulation cascade. In a study consisting of 421 people, authors demonstrated that subjects affected by FH with a lack functional LDLR had increased levels of FVII suggesting that the *LDLR* might have a suppressing role on this glycoprotein blood levels [36]. Additionally, *LDLR* locus single nucleotide polymorphisms were linked to CAD, independent of lipid profile and consistent with FVII levels. The *LDLR* locus may have pleiotropic influences on either plasma lipid or coagulation factor levels, which in turn may modulate the risk of CVD [37].

Besides, based on current literature, lowering lipids drug are suspected to be related to coagulation cascade. Statins seem to have a pleiotropic effect through thrombotic factors [38], meanwhile *PCSK9* may play an interesting role in the process of thrombogenesis. It was observed that *PCSK9* knockout mice have reduced platelet activity and developed less agonist-induced arterial thrombosis compared to the animal control group. Otherwise, elevated blood levels of PCSK9 in humans are associated with an increased platelet reactivity [39]. The breakdown of this blood clotting factor is facilitated by LDLR [40] and PCSK9 inhibitor decreased plasma levels/activity of fibrinogen and plasminogen activator inhibitor 1 [41]. As a result, PCSK9 raises FVII. To conclude, the coagulation factors may play a critical role in FH [42]; however, their mechanisms are still unclear.

Lp(a) is a macromolecular complex made up of one LDL-particle molecule covalently bound to an apoB-100 containing polymorphic glycoprotein molecule apo(a) [43]. Apo(a) is characterized by a triple loop-like structure called “Kringle”, similar to other coagulation factors such as plasminogen (PLG) or prothrombin. Lp(a) blocks the plasmin formation competing with PLG for the binding sites on endothelial cells [44]. This process leads to a delay in fibrinolysis, which means an increased ratio of thrombosis [45]. Despite its role in venous thromboembolism, Lp(a) is linked to an increased incidence of CVD and in particular coronary heart disease [46,47]. Lp(a) is also an independent risk factor for premature cardiovascular events in the general population due to a pro-atherosclerotic effect of ApoB-100. Its blood concentration is significantly influenced by sex, gender, lifestyle and chronic diseases and it seems that hyperlipoproteinemia (a) enhances the occurrence of atherosclerotic cardiovascular diseases in FH [48]. Alonso et al. studied a population of over than 2000 patients, which includes individuals with or without FH, and it has been shown that FH patients, especially those with history of CVD, had elevated Lp(a) plasma levels. A significant high plasma levels of Lp(a) has been related to null and defective mutations of LDLR. Indeed, the Lp(a) could be considered an independent predictor of cardiovascular disease [49]. Furthermore, higher blood levels of Lp(a) in individuals affected by FH are linked to an elevated occurrence of Lp(a) variants and the risk of CVD is increased twofold when both conditions coexist [50]. Hyperlipoproteinemia(a) has been proven to be a predictor of premature atherosclerotic cardiovascular disease in a cohort of patients affected by FH [51]. Furthermore, the measured blood levels of LDLc consist of aggregating LDL and Lp(a) particles. The cholesterol content of Lp(a) constitutes up to 30–45%; therefore, it is necessary to correct LDLc in order to determine the proportion of Lp(a). A study including more than 500,000 individuals discovered that LDLc was no longer a risk factor for incident cardiovascular disease if the correction of LDLc was implemented [52]. Additionally, high blood levels of Lp(a) lead to elevated risk of myocardial infarction [53]; therefore, a routinely testing of Lp(a) may identify individuals who will develop more premature cardiovascular events [54].

**Table 2:** Familial hypercholesterolemia, coagulation factors and lipoprotein a.

Authors	Type	Year	Subjects	Findings
Narverud et al. [32]	Observational	2013	16 children born of mother with FH compared to 16 ones born of mother without FH	High level of plasminogen activator inhibitor and tissue factor pathway inhibitor in children born from mother affected by FH independently of children FH status
Bianciardi et al. [33]	Comparative	2015	11 type 2 diabetic patients, 6 FH patients and 5 healthy subjects	Platelet activation is increased in people affected by FH and type 2 diabetes
Icli et al. [34]	Mulricenter study	2016	324 individuals, 164 affected by FH and 160 without	MPV was increased in patients affected by FH compared to the control
Huijgen et al. [36]	Comparative	2011	421 individuals both affected by FH and non-affected were enrolled	FH group with a lack functional LDLR had increased levels of FVII suggesting that the LDLR might have a suppressing role on this FVII
Martinelli et al. [37]	Comparative	2010	1122 patients undergoing a coronary angiography examination	Pleiotropic effect of LDLR locus on plasma lipid and coagulation factor levels, and its role in modulating CVD risk
Alonso et al. [49]	Observational	2014	1960 patients with FH and 957 non-FH	Lpa is an independent predictive risk factor for CVD in people affected by FH. Lp(a) levels > 50 mg/dL and negative mutation in the LDLR increase CVD risk
Page et al. [51]	Comparative	2020	763 patients affected by FH	Lp(a) rs3798220-C allele is associated with FH and Lp(a) high levels are predictive factors of coronary artery disease
Ellis et al. [54]	Comparative	2019	2927 people tested for genetic FH and Lp(a) levels > 50 mg/dL	Individuals affected by FH with high Lp(a) blood levels have an increased atherosclerotic cardiovascular risk

Lp(a) = lipoprotein (a); CVD = cardiovascular disease; LDLR = low density lipoprotein receptor; FH = Familial Hypercholesterolemia.

### 3.3. Unexpected Role of the Microbiome in Familial Hypercholesterolemia

The gut microbiota consists of trillions of microorganisms inhabiting the human intestine, which comprise bacteria, archaea, eukarya, viruses and parasites, exerting significant influence on both physical and mental health of the host. More recently, the focus has been extended to the role of the gut microbiota in CVD and there still is no definitive understanding of what truly constitutes a healthy adult gut microbial profile. Generally, the gut microbiota consists of six phyla including *Firmicutes*, *Bacteroidetes*, *Actinobacteria*, *Proteobacteria*, *Fusobacteria*, and *Verrucomicrobia* [55]. Among these seven divisions, *Firmicutes* and *Bacteroidetes* are the major types (more than 90% of the total population). The human gut microbiota also contains viruses, phages, and archaea, mainly *M. smithii* and fungi (the most studied are *Candida, Saccharomyces*, *Malassezia, and Cladosporium*) [56]. Furthermore, the distribution of gut microbiota varies in different anatomical parts of the gastrointestinal tract [57]. The small intestine (duodenum and jejunum), due to the short transit time and the high presence of bile acids, has the lower richness and abundance of colonizers (from 10^3^ to 10^5^ CFU/mL), while the colon shows the largest microbial community (10^12^ CFU/mL), composed prevalently of anaerobic microorganisms, as a result of the slow flow rate, pH ad O_2_ tension, nutrient-poor environment [58].The gut microbiota has been shown to be a central regulator of host metabolism and it is closely involved in vitamin and amino acid biosynthesis, bile acids biotransformation, dietary fibre fermentation, and short chain fatty acids production [55]. The gut microbiota and its metabolites such as SCFA, lipopolysaccharides (LPS), and trimethylamine N-oxide (TMAO) may affect atherosclerosis, through different mechanisms [22,59,60]. In particular, the microbiota-derived choline-metabolite TMAO is strongly associated with CVD [61] and acute coronary syndrome at a very young age [62]. It originates from a substance known as trimethylamine (TMA), which is primarily released by the *Clostridia* and *Enterobacteriaceae* families, during the degradation of nutrients such as carnitine, choline, and lecithin, which are found in meat and egg. Hepatic flavin monooxygenases then oxidize trimethylamine into TMAO. Current evidence suggests that it plays a key role in the pathogenesis of atherosclerosis, promoting endothelial dysfunction, increasing atherosclerotic plaque size and plaque instability, triggering prothrombotic platelet function, and promoting arterial thrombus growth [63,64,65]. Circulating levels of TMAO positively correlate with the incidence of major cardiovascular events, and are prognostic for an adverse outcome, including death, in ACS and periphery artery disease (PAD) patients [66]. Because TMAO promotes platelet hyper-reactivity and uptake of oxidized-LDLc by macrophages (through the production of SR-A1 and CD36), its heightened levels are linked to an increase in the expression of pro-inflammatory cytokines [67]. In addition, it seems to downregulate the anti-inflammatory IL-10 and nitric oxide (NO) expression and to increase reactive oxygen species (ROS) generation, then favouring endothelial dysfunction [65,68,69]. It is demonstrated that TMAO enhances stimulus-dependent Ca^2+^ release from intracellular reserves, hence promoting platelet aggregation and thrombus formation [23] and can hasten the atherosclerotic processes by interfering with lipid metabolism, particularly cholesterol metabolism [70] The findings of a cohort study of patients with CAD revealed that TMAO levels were significantly associated with plaque vulnerability to rupture [71]. The plaque might be considered a microbial environment in and of itself, containing microbes such as *Streptococcus*, *Pseudomonas*, *Klebsiella*, *Veillonella* spp., and *Chlamydia pneumoniae* [59,72]. The bacterial colonization of the atherosclerotic plaque may affect its composition [65]. In addition, different studies were unable to link the composition of the plaque microbiota to plaque vulnerability, rupture, or cardiovascular events [73,74]. Otherwise, the oral and gut microbiota components could affect plaque formation and vulnerability via indirect mechanisms, involving vessel wall pathobiology. An augmented gut permeability, allowing the translocation of the bacterial components, may cause cytokines release and an inflammatory condition that could induce or exacerbate atherogenic processes. Moreover, it is conceivable that numerous potential homologous pathogen/host peptides could trigger an autoimmune response (molecular mimicry) contributing to vessel wall damage and atherogenesis [75]. Gut bacteria such as *Dysgonomonas*, *Paraprevotella*, *Succinatimonas*, and *Bacillus* are associated with elevated inflammatory or prothrombotic biomarkers (interleukin-6, fibrinogen, homocysteine, C reactive protein) and with a greater plaque volume [76]. A lower abundance of *Lachnospiraceae NK4B4* group, *Lachnospiraceae UCG-004*, and *Ruminococcus Gauvreauii*, and a higher abundance of *Ruminococcus gnavus* are significantly associated with CAD in people affected by dyslipidaemia and diabetes mellitus [77]. Recently, Curini et al. have also documented the presence of a valve microbiota in patients affected by calcific aortic valve disease (CAVD), encouraging to exploration of the link between bacteria and the immune system and its role in the valve calcification process [78]. Gut microbiota may contribute to atherogenesis, despite plasma lipids and TMAO, through inflammatory pathways. Hence, the gut microbiota is a very important contributing factor in hyperlipidemia and CVD, but its role in patients affected by FH is still elusive. Evidence regarding the role of gut microbiota and its metabolites in acute coronary syndromes and FH are summarized in Table 3. It was shown that the presence of a pro-inflammatory microbiota derived from Caspase1^−/−^ mice is sufficient to promote atherosclerosis in antibiotic-treated *Ldlr*^−/−^ mice [79]. The pathophysiology of CVD comprises a chronic low-grade inflammation that may be caused by oral and gut microbiota. A cohort of patients with CVD showed a significantly greater *Porphyromonas gingivalis* abundance compared to healthy subjects and heterozygous FH subjects in primary prevention [80]. Additionally, in a family affected by FH with a history of myocardial infarction that occurred at a very young age, the intestinal *Prevotella dentalis* was found elevated and strongly associated with LDLc levels in ones with mutation of *LDLR* compared to the unaffected members with hyperlipidemia [81]. Christopher Storm-Larsen et al. found additional intriguing evidence when they compared the gut microbiota composition of FH patients taking lipid-lowering medications to that of healthy controls. They found a marked decrease in bacterial communities as well as a decline in some bacterial taxa including *Clostridia* and *Coriobacteriia* in FH patients compared to the controls. However, they were not able to discern whether gut microbiota changes were secondary to FH or a drug treatment result [82].

The relationship between the gut microbiota and FH is still poorly defined, but the microbiota may play a role in affecting inflammatory responses and lipid metabolism. Intriguingly, Pasariello et al. showed the effectiveness of a symbiotic containing *Lactobacillus paracasei* B21060 in lowering lipid biomarkers in kids with FH in a prospective, randomized, case–control study [83].

### 3.4. New Potential Therapeutic Strategies for Familial Hypercholesterolemia

The intriguing role of microbiota in atherosclerosis and cardiovascular diseases opens the way for new potential treatments targeting gut microbiota composition or microbial-derived metabolites also for FH treatment.

Several cutting-edge treatment strategies that target TMAO production and metabolism are under investigation. A proposed strategy for the reduction in TMAO relies on the blocking of precursor production; 3,3-Dimethyl-1-butanol (DMB), a structural analogue of choline, can decrease the production of TMA by reducing TMA-producing intestinal bacteria or directly inhibiting microbial choline TMA lyase activity [84]. Further, the iodomethylcholine (IMC) and fluoromethylcholine (FMC) molecules, novel choline TMA lyase inhibitors, can reduce the host’s TMA and TMAO levels leading to the suppression of platelet aggregation and the in vivo rate of thrombus formation without increased bleeding time [85] and promoting a favourable reorganization of host cholesterol and bile acid metabolism [86].

Overall, less consumption of choline-rich foods and physical exercise could represent a strategy to reduce TMAO levels (Erickson ML, Malin SK, Wang Z, Brown JM, Hazen SL, Kirwan JP. Effects of lifestyle intervention on plasma trimethylamine N-oxide in obese adults). According to Ivashkin et al., lower consumption of L-carnitine and phosphatidylcholine-containing foods reduces TMAO plasma concentration in CAD patients, probably through a modification of the gut microbiota composition [87]. Indeed, specific bacterial taxa in human faeces, such as *Clostridia* and *Peptostreptococcaceae* [88], have been associated with both plasma TMAO concentration and dietary status.

Another strategy to lessen the occurrence of atherosclerosis in FH patients could be the regulation of gut microbial composition with probiotics/prebiotics supplementation or faecal microbiota transplantation (FMT). Probiotic strains such as Lactobacillus acidophilus, Bifidobacterium longum, and Lactobacillus plantarum seem to have hypocholesterolemic properties [89]. Dietary supplementation with *L. acidophilus*, *B. bifidum*, and oligofructose increases the HDL-c level, reduces CVD risk, and significantly reduces the blood sugar level in elderly people with type 2 diabetes mellitus [90]. In a randomized double-controlled trial on hypercholesterolemic patients, *L. plantarum* strains improved cholesterol profile and reduced CVD risk [91]. Anyway, probiotics’ efficacy is sometimes objectionable, since it is considered strain- and disease-dependent [92]. To really understand their efficacy, well-designed trials, with untargeted microbiota sequencing (i.e., shotgun) before and after the intervention, should be performed. Indeed, defining treatment effects on gut microbiota functionality, other than on taxonomic composition, is essential.

Finally, FMT could be used to optimize the gut microbiota composition in FH individuals at risk for cardiovascular events. To date, the results of an FMT trial with transplantation of faeces from lean vegan subject to metabolic syndrome patients have resulted in microbiota alteration without changing TMAO levels [93]. Indeed, the optimal FMT approaches, including donor selection, and treatment strategies (number of sessions and times), have yet to be defined [94,95].

## 4. Conclusions

LDLc has been considered the leading cause of premature cardiovascular events in individuals affected by FH. New factors are now understood to have an intriguing role in the development of atherosclerotic diseases in FH patients. The study of the human gut microbiota has greatly increased in recent years. Alterations in gut microbiota composition and functions have been demonstrated to have a direct effect on human health and to play an important role in the occurrence of several diseases including CVD. Although the data suggest that microbiome dysbiosis might cause cardiovascular disorders in higher risk patients as well as those with FH, there are still significant knowledge gaps in the relationship between the gut microbiota and the host. Ergo, there are still many unanswered questions about it. The research of the microbiota is also mostly concentrated on the bacterial component and the function of fungi or viruses, whereas other microorganisms are yet largely unexplored. In our opinion, randomized controlled trials, with several investigations over time and with an untargeted analysis of microbiome, before and after intervention, are the only way to learn more about how the gut microbiota influences the complex pathophysiology of human FH.

## Figures and Tables

**Table 1:** Association between acute coronary syndromes and Familial Hypercholesterolemia.

Authors	Type	Year	Subjects	Findings
Cao et al. [25]	Observational	2018	105 patients with very early-onset CAD	The prevalence of FH pathogenic mutations was 38.1%
Kheiri et al. [26]	Observational	2021	1,697,513 ACS admissions to the hospitals	People affected by FH were younger and more likely to present ST elevation and high incidence of recurrence among people with ACS
Singh et al. [27]	Retrospective	2019	1996 patients with a median age of 45 years	1 in 10 patients with myocardial infarction at young age was affected by FH
Rallidis et al. [28]	Prospective	2016	320 individuals who had their first STEMI ≤ 35 years of age	1 in 5 patients have clinical heterozygous FH and high recurrence rate of cardiac events during long-term follow-up
Danchin et al. [29]	Observational	2020	5147 patients admitted to the hospital and discharged alive (FH status not known)	risk of long-term mortality is twice as high in FH population

FH = Familial Hypercholesterolemia; CAD coronary artery disease; ACS = acute coronary syndrome.

**Table 3:** The gut microbiota and its metabolites in acute coronary syndromes and familial hypercholesterolemia.

Authors	Type	Year	Subjects	Findings
Taşcanov et al. [62]	Observational	2020	127 individuals affected by acute coronary syndromes	Young patients who have developed acute coronary syndromes had higher levels of TMAO and choline compared to the elderly group
Curia et al. [80]	Observational	2022	30 patients without HeFH and 10 patients with HeFH	The heterozygous FH subgroup showed a *Porphyromonas gingivalis* abundance even greater than that of non-heterozygous FH patients with CVD.
Liu et al. [81]	Observational	2021	Three-generation Chinese family with FH	The gut microbiota in FH individuals may exhibit stronger function of pro-inflammatory potential which facilitates lipometabolic disturbance.
Storm-Larsen et al. [82]	Retrospective	2022	Heterozygote FH aged 18–75 and using statins > 12 months	Individuals with FH have an altered gut microbial composition especially individuals on cholesterol-lowering drug ezetimibe in addition to statins.
Brandsma et al. [79]	Observational	2019	female *Ldlr*^−/−^ mice transplanted with fecal microbiota from *Casp1*^−/−^ mice	Pro-inflammatory microbiota is sufficient to promote atherosclerosis in antibiotic-treated *Ldlr*^−/−^ mice

FH = familial hypercholesterolemia; LDLR = low-density lipoprotein receptor; CVD = cardiovascular disease; TMAO = trimethylamine-N-oxide.

## Data Availability

No new data were created or analyzed in this study. Data sharing is not applicable to this article.
